# MicroRNA-31 Is Overexpressed in Cutaneous Squamous Cell Carcinoma and Regulates Cell Motility and Colony Formation Ability of Tumor Cells

**DOI:** 10.1371/journal.pone.0103206

**Published:** 2014-07-28

**Authors:** Aoxue Wang, Ning Xu Landén, Florian Meisgen, Warangkana Lohcharoenkal, Mona Ståhle, Enikö Sonkoly, Andor Pivarcsi

**Affiliations:** Unit of Dermatology and Venereology, Department of Medicine, Karolinska Institutet, Stockholm, Sweden; The University of Tennessee Health Science Center, United States of America

## Abstract

Cutaneous squamous cell carcinoma (cSCC) is a malignancy of epidermal keratinocytes that is responsible for approximately 20% of skin cancer-related death yearly. We have previously compared the microRNA (miRNA) expression profile of cSCC to healthy skin and found the dysregulation of miRNAs in human cSCC. In this study we show that miR-31 is overexpressed in cSCC (*n* = 68) compared to healthy skin (*n* = 34) and precancerous skin lesions (actinic keratosis, *n* = 12). LNA *in situ* hybridization revealed that miR-31 was specifically up-regulated in tumor cells. Mechanistic studies of inhibition of endogenous miR-31 in human metastatic cSCC cells revealed suppressed migration, invasion and colony forming ability, whereas overexpression of miR-31 induced these phenotypes. These results indicate that miR-31 regulates cancer-associated phenotypes of cSCC and identify miR-31 as a potential target for cSCC treatment.

## Introduction

Cutaneous squamous cell carcinoma (cSCC) is the second most common skin cancer among fair-skinned people, with an increasing incidence over the last decades. cSCCs are associated with a substantial risk of metastasis and responsible for approximately 20% of skin cancer-related death yearly [1], [2]. cSCCs can develop on precancerous lesions such as actinic keratosis (AK) or Bowen’s disease, and the risk to develop cSCCs is strongly associated with chronic sun (ultraviolet light) exposure [3]. Other known environmental risk factors for cSCC include ionizing radiation, cigarette smoking, and certain chemical exposures such as arsenic. Induced or acquired immunosuppression after organ transplantation or in patients diagnosed and treated for leukemia are also recognized as significant risk factors for the development of cSCC, and these tumors portend a worse prognosis with twice the risk of developing metastasis compared to immunocompetent patients [4]. Numerous pathways are reported to be involved in cSCC development, including mutation or UVB-induced inactivation of p53, amplification and activating mutations of the Ras oncogene and NF-κB activation [2], [5], but only few studies have investigated the role of microRNAs (miRNAs) in this cancer type [6], [7].

MiRNAs are a class of short, non-coding RNA molecules which regulate gene expression at the post-transcriptional level [8]. MiRNAs are involved in the regulation of a variety of biological processes including cell cycle, differentiation, development, and metabolism. Deregulation of miRNA expression has been observed in cancer, in which miRNAs can act as tumor suppressors or oncogenes depending on the tissue and the set of targets they regulate [9]. For example, miR-29 family members were shown to function as tumor suppressors and their down-regulation being associated with the development and progression of several human malignancies including lung cancer, invasive breast cancer and hepatocellular carcinoma. In contrast, miR-21 functions as an oncogene and overexpression of this miRNA has been observed in nearly all human malignancies and associated with important cancer hallmarks, such as uncontrolled cell proliferation, decreased apoptosis, invasion, and migration [10]. Moreover, some miRNAs, for example, miR-31, can behave as either tumor suppressor or oncogenic miRNA depending on the tissues they are located [11]. Strikingly, the survival of certain tumors can be completely dependent on the expression of specific oncogenic miRNAs (oncomiRs), for example, inactivation of miR-21 or miR-155 in tumors overexpressing these miRNAs can lead to complete regression of these tumors in mice [12]. Thus, restoration or silencing of cancer-associated miRNAs could lead to a favourable phenotype and may be used as a therapeutic approach in cSCC.

We have previously identified the dysregulation of miRNAs in human cSCC compared to healthy skin by miRNA expression profiling and demonstrated that hierarchical clustering based on miRNA expression could clearly separate cSCC tumors from healthy skin samples [7]. The majority of miRNAs with significantly changed expression were down-regulated in cSCC. Interestingly, 4 miRNAs were found to be up-regulated in cSCC and among these, miR-31 was the most up-regulated [7]. Since miR-31 was described as a master regulator of metastasis and has become obvious that it does play a major role in regulating several other cancer associated cellular characteristics as well [13], we set out to determine the potential roles of miR-31 in the regulation of cancer associated phenotypes including cell migration, cell invasion and colony formation of cSCC.

## Materials and Methods

### Clinical Samples

Punch biopsies were obtained and snap-frozen, after written informed consent, from skin of healthy donors (*n* = 21), and from skin lesions of patients with actinic keratosis (AK, *n* = 11) and cSCC (*n* = 13) at the Dermatology and Venereology Unit, Karolinska University Hospital, Stockholm, Sweden and at the Department of Dermatology, Heinrich Heine University, Düsseldorf, Germany. The clinical diagnosis was made by a dermatologist and confirmed by histopathological evaluation. The formalin-fixed, paraffin embedded (FFPE) normal skin (*n* = 13) and stage I-III cSCC biopsies (*n* = 55) were obtained from Karolinska University Hospital Biobank. The study was approved by the Regional Ethics Committees (Duesseldorf, Germany and Stockholm, Sweden) and conducted according to the Declaration of Helsinki Principles. RNA was extracted from frozen biopsies or formalin-fixed, paraffin-embedded tissue sections using the method described previously [14] and miRNeasy FFPE Kit (Qiagen, Sollentuna, Sweden), respectively.

### Quantitative Real Time PCR

Quantification of miR-31 was carried out by TaqMan Real-Time PCR according to the manufacturer’s instructions (Life Technologies, Stockholm, Sweden). Briefly, 10 ng of template RNA was reverse transcribed using the TaqMan MicroRNA Reverse Transcription Kit and miR-31-specific stem-loop primers (Life Technologies, assay ID: 002279). RT product at 1.5 µl was introduced into the 20 µl PCR reactions which were incubated in 384-well plates on the ABI 7900HT thermocycler (Applied Biosystems) at 95°C for 10 min, followed by 40 cycles of 95°C for 15 s and 60°C for 1 min. MiR-31 expression was normalized between different samples based on the values of U48 small nucleolar RNA expression (Life Technologies, assay ID: 001006).

### *In Situ* Hybridization

*In situ* hybridization was performed on formalin-fixed paraffin-embedded sections (10 µM thickness) of skin biopsy specimens. Briefly, after incubation in acetylation solution (0.06 M HCl, 1.3% trietanolamin, and 0.6% acetic anhydride in filtered water) for 10 min at room temperature, sections were incubated in permeabilization buffer (1% of Triton X-100) for 30 min at room temperature, washed, and prehybridized for 1 h at 50°C. Hybridization with digoxigenin (DIG)-labeled miRCURY locked nucleic acid (LNA) probes (Exiqon, Vedback, Denmark) was performed over night at 50°C. Slides were then washed four times with 2×SSC buffer followed by one time with 0.1×SSC buffer at 67°C. The probe binding was detected by incubating the sections with alkaline phophatase-conjugated sheep anti-digoxigenin Fab fragments (Roche, Stockholm, Sweden) at 1∶2500 dilution for 1 h at room temperature. Sections were visualized by adding BM purple alkaline phophatase substrate (Roche), according to the manufacturer’s instructions.

### Cell Culture and Transfections

Human metastatic cutaneous SCC cells (UT-SCC-7), a kind gift from Professor Veli-Matti Kähäri, Turku, Finland [15] were grown under humidified atmosphere of 5% CO_2_ at 37°C in Dulbecco’s modified Eagle’s medium (DMEM) supplemented with 10% fetal bovine serum (FBS), 10 mM 4-(2-hydroxyethyl)-1-piperazineethanesulfonic acid (HEPES), 100 µM MEM Non-Essential Amino Acids Solution and 100 units/ml penicillin/streptomycin (PEST) (Life Technologies, Stockholm, Sweden). For functional studies, cells were transfected with 10 nM miR-31 miRNA precursor (Pre-miR-31) or miRNA precursor negative control #1 (Scramble) (Ambion) and with 10 nM miRIDIAN miR-31 hairpin inhibitor or miRNA hairpin inhibitor negative control #1 (Scramble) (ThermoFisher Scientific, Västra Frölunda, Sweden) using Lipofectamine 2000 (Life Technologies, Stockholm, Sweden) following the manufacturers' instruction. Overexpression and inhibition of the mature, biologically active form of miR-31 was confirmed by real-time PCR after transfection.

### Scratch-Wound Assay

Transfected cells were grown to confluence and a scratch was made with a sterile 200 µl pipette tip. The cells were kept in medium containing 5% FBS and photographed at 0 hour and 7 hours after scratch. The area of the wound was determined with ImageJ. Wound closure rate = (area of the wound at 0 h - area of the wound at 7 h)/area of the wound at 0 h.

### Migration and Invasion Assays

Cell migration and invasion were determined in the 24-well plate Transwell system with 8 µm pore size polycarbonate filter and BD Matrigel invasion chamber (BD Biosciences, NJ). Briefly, cells at the density of 2.5×10^4^ cells per well were seeded into the upper chamber of the Transwell unit in serum-free medium. The lower chamber of the unit was added with a normal growth medium containing 10% FBS. The unit was incubated at 37°C in a 5% CO_2_ atmosphere for 24 or 48 h. The non-migrating or non-invading cells were removed from the inside of the insert with a cotton swab. Cells that migrated or invaded to the lower side of the membrane were stained with crystal violet (Sigma-Aldrich) and visualized under a light microscope. The number of migrating/invading cells were counted in 10 randomly selected fields and the average number of was calculated in each sample. This experiment was performed three times independently and the data of one representative experiment are shown.

### Soft Agar Colony Formation Assay

UT-SCC-7 cells were seeded at 4000 cells/cm^2^ in 24 well plates in DMEM supplemented with 10% FBS, 100-units/ml PEST and 0.3% agarose with 0.6% agarose underlay. Dishes were incubated at 37°C in a humidified atmosphere containing 5% CO_2_. After 10 days, colonies were stained with crystal violet (Sigma-Aldrich) and counted.

### Statistical Analysis

Statistical significance for experiments was determined by Mann-Whitney U Test or Student’s *t-*test.

## Results

### Up-regulation of miR-31 in skin squamous cell carcinoma

**Figure 1:**
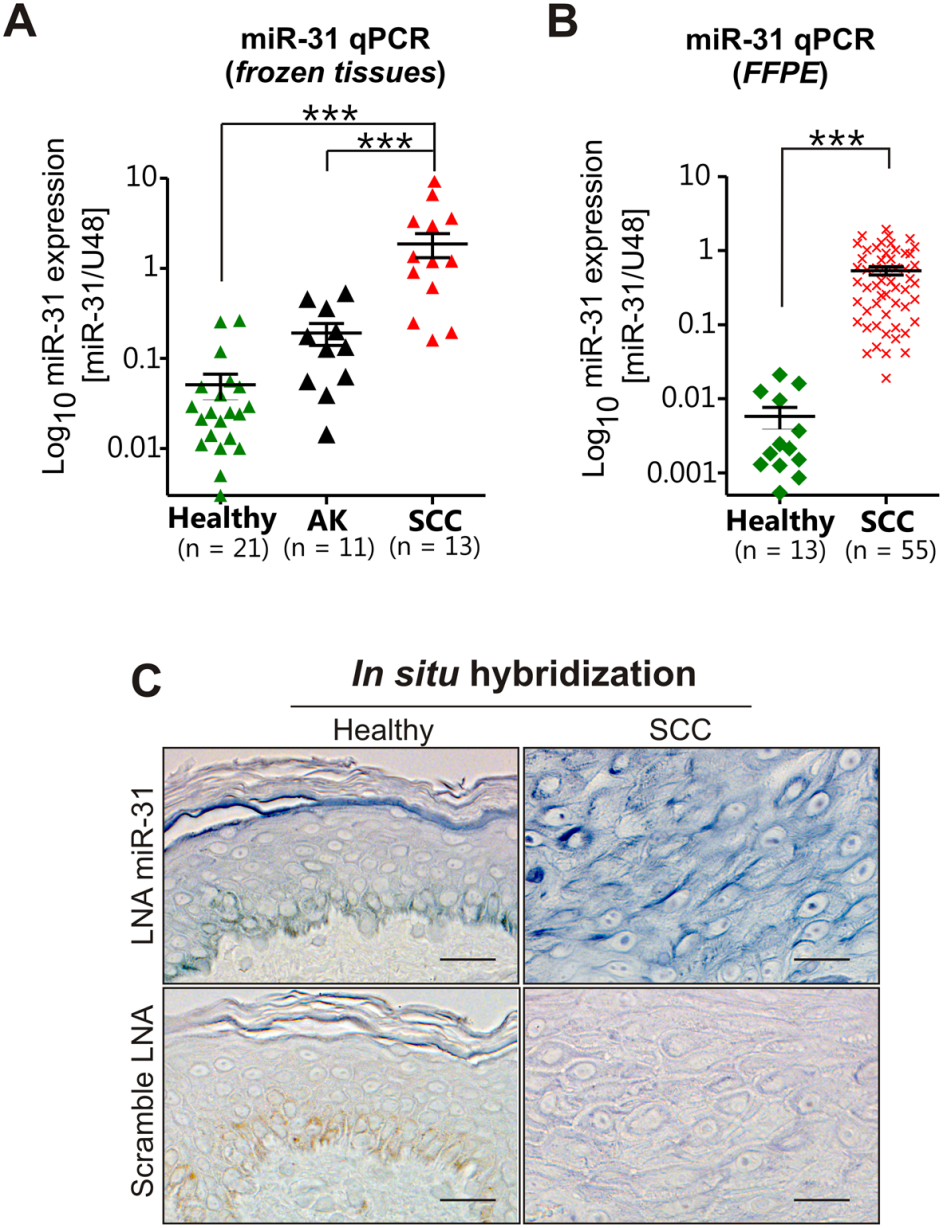
MiR-31 is overexpressed in cutaneous squamous cell carcinoma. (A) qPCR analysis of miR-31 in healthy skin, actinic keratosis lesion and cSCC in fresh-frozen clinical material. ***p<0.001, Mann-Whitney U-test. (B) qPCR analysis of miR-31 levels in archived, formalin-fixed paraffin-embedded healthy skin and cSCC. ***p<0.001, Mann-Whitney U-test. (C) In situ hybridization (ISH) was performed on a normal skin and skin SCC sections using DIG-labeled locked nucleic acid (LNA)-based probes for miR-31. Scrambled LNA-probe was used as control. Representative micrographs for miR-31 expression in healthy skin (Healthy) and cSCC tissues (SCC) are shown. Scale bar: 50 µm.

Recently, we observed the deregulation of miRNA expression in cSCC and identified miR-31 as the most up-regulated miRNA in cSCC compared with healthy skin as determined by miRNA array [7]. To validate the array data and to explore the expression of miR-31 during skin cancer progression, we performed quantitative real-time PCR (qRT-PCR) on a set of RNA isolated from fresh-frozen skin biopsies obtained from healthy skin (*n* = 21), actinic keratosis (AK, pre-cancerous skin lesions, *n* = 12), and cSCCs (*n* = 13). We demonstrated that miR-31 is significantly up-regulated in cSCC as compared to healthy skin and AK lesions (*p*<0.001 for both) (Figure 1A). Interestingly, miR-31 was not overexpressed in AK compared to healthy skin, suggesting that the up-regulation of miR-31 occurs late during tumorigenesis when lesions become invasive. Similarly, qRT-PCR analysis of miR-31 expression in an independent set of formalin-fixed, paraffin embedded (FFPE) samples, including 13 healthy skin biopsies and 55 cSCCs showed significant overexpression of miR-31 in cSCC (*p*<0.001, Figure 1B). To examine whether overexpression of miR-31 in cSCC is due to its up-regulation in tumor cells, or the altered cellular composition of the tumor e.g. due to infiltration of immune cells, we performed *in situ* hybridization on healthy skin (n = 6) and cSCC (n = 5) specimens. MiR-31 was highly expressed in cSCC cells but had low expression in healthy epidermal cells (Figure 1C). These results suggested the involvement of miR-31 in cutaneous squamous cell tumorigenesis.

### Induction of cSCC motility by miR-31

**Figure 2:**
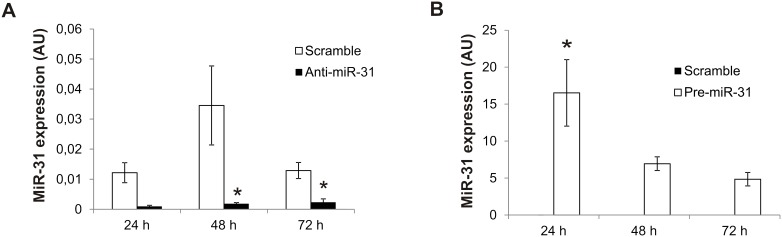
Transfection efficiency of anti-miR-31 and pre-miR-31 represented as miR-31 expression level. (A) UT-SCC-7 cells were transfected with anti-miR-31 or control oligonucleotides (scramble) and the expression level of miR-31 was detected at 24, 48 and 72 h after the transfection by PCR. (B) UT-SCC-7 cells were transfected with pre-miR-31 or control oligonucleotides (scramble) and the expression level of miR-31 was detected at 24, 48 and 72 h after the transfection by PCR. The data of one representative experiment performed in triplicates are shown and bars depict mean±SD. The experiment was repeated three times. *p<0.05. Student’s t-test.

**Figure 3:**
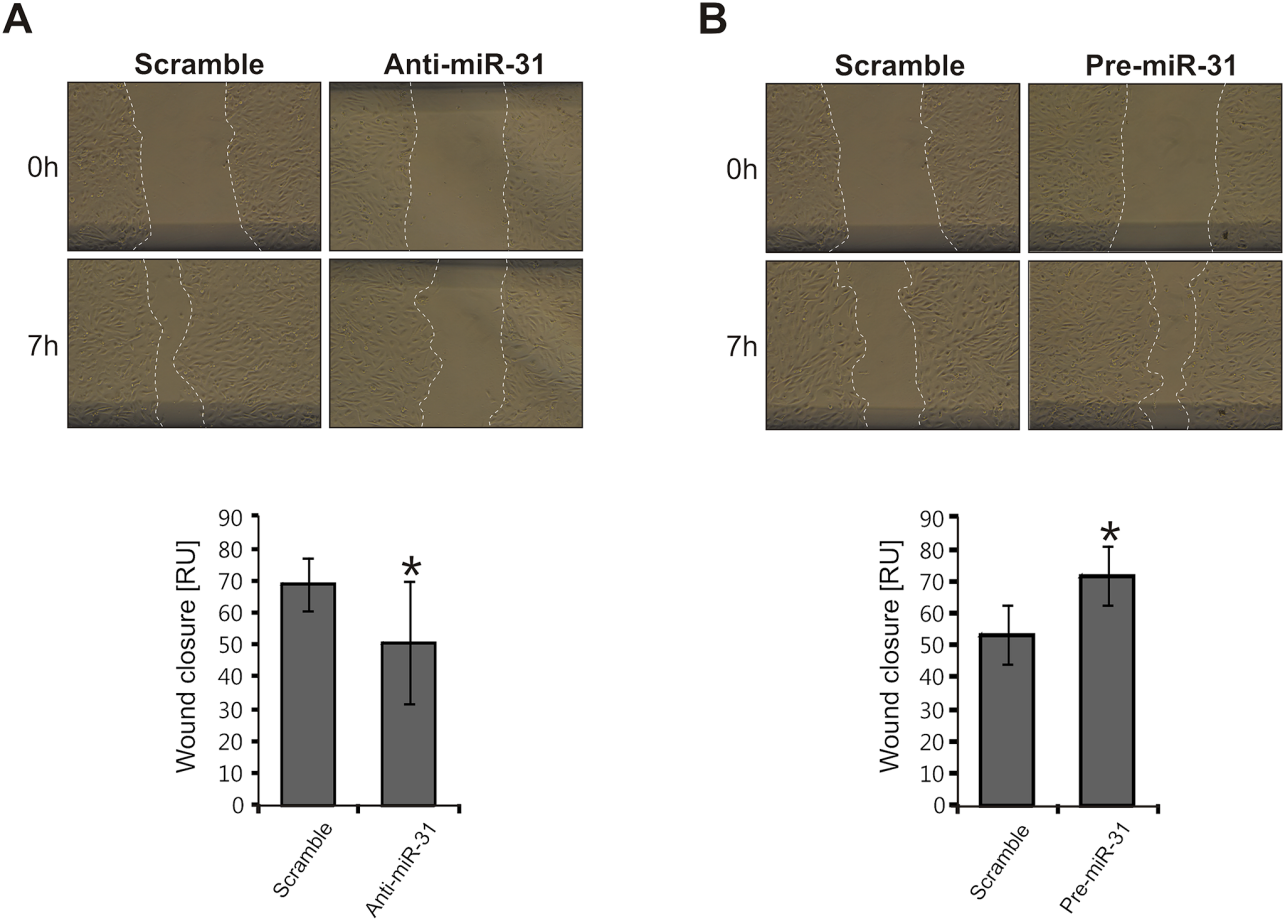
MiR-31 promotes motility of cSCC cells. Scratch-wound assay was performed to assess the migration rate of UT-SCC-7 transfected with (A) miR-31 inhibitors (anti-miR-31) or (B) synthetic miR-31 (pre-miR-31) compared to control oligonucleotides (scramble). Photographs were taken at indicated time points after scratch injury. The migration rates of UT-SCC-7 were quantified by measuring the area of the injured region. Data of one representative experiment performed with six replicates are shown and this experiment was repeated three times. The bar chart depict mean ± SD of relative wound closure. *p<0.05, Student’s t-test.

**Figure 4:**
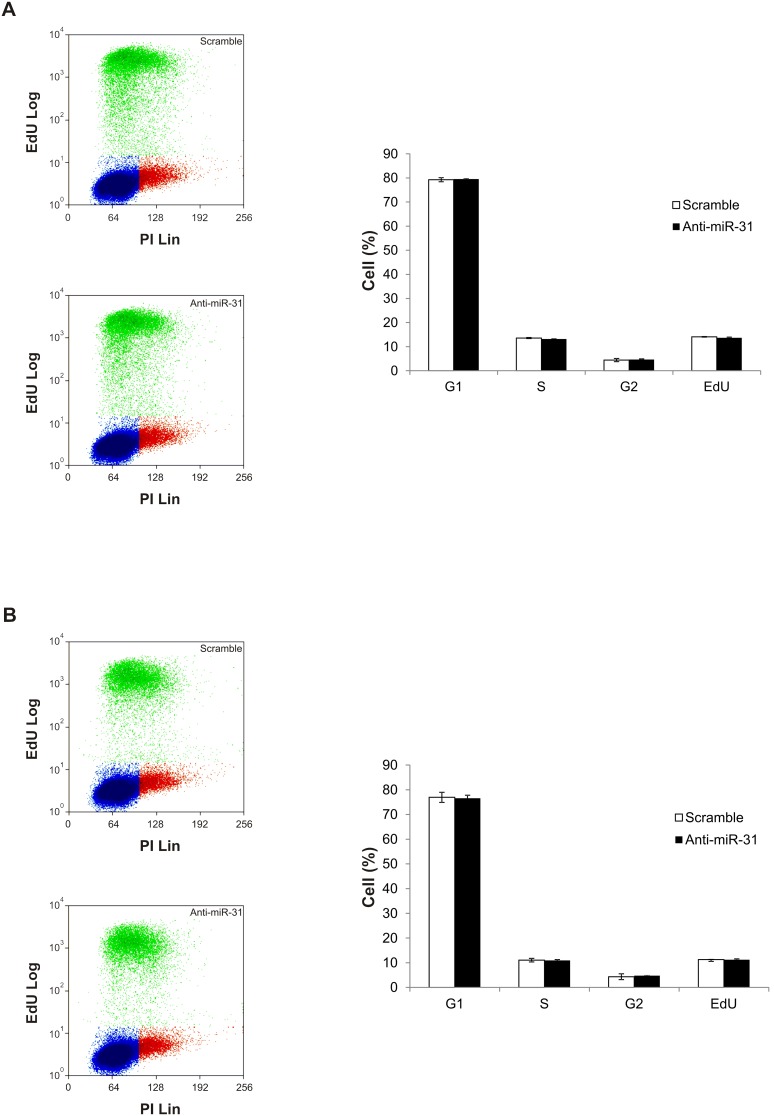
Inhibition of miR-31 does not affect cSCC proliferation and cell cycle. UT-SCC-7 cells were transfected with anti-miR-31 or control oligonucleotides (scramble) and subsequently determined for cell proliferation and cell cycle progression by FACS at 48 (A) and 72 h (B) after the transfection using EdU flow cytometry assay. Bars depict mean ± SD of the three independent experiments: percentage of cells in the G1, S, G2-phase of the cell cycle and percentage of EdU+ cells are shown.

To investigate the role of miR-31 in cSCCs cells, we overexpressed miR-31 or inhibited endogenous miR-31 in a human metastatic cutaneous SCC *cell line*, UT-SCC-7, by transfecting miR-31 precursors (Pre-miR-31) or inhibitors (Anti-miR-31) respectively. Overexpression and inhibition of the mature, biologically active form of miR-31 was confirmed by real-time PCR after transfection (Figure 2). In order to determine the effect of miR-31 expression on cSCC motility, we have performed scratch assays. As shown in the representative photographs and calculated wound area in bar charts, inhibition of endogenous miR-31 significantly reduced the rate of wound closure of UT-SCC-7 cells in comparison with control LNA-treated tumor cells (Figure 3A). Moreover, overexpression of miR-31 accelerated this process (Figure 3B). We found that inhibition of miR-31 in UT-SCC-7 cells did not change the rate of cell proliferation, indicating that the effect of miR-31 on wound closure was not due to its effect on cell proliferation (Figure 4). These results suggest that miR-31 regulates the motility of cSCC cells.

### MiR-31 Promotes cSCC migration and invasion

**Figure 5:**
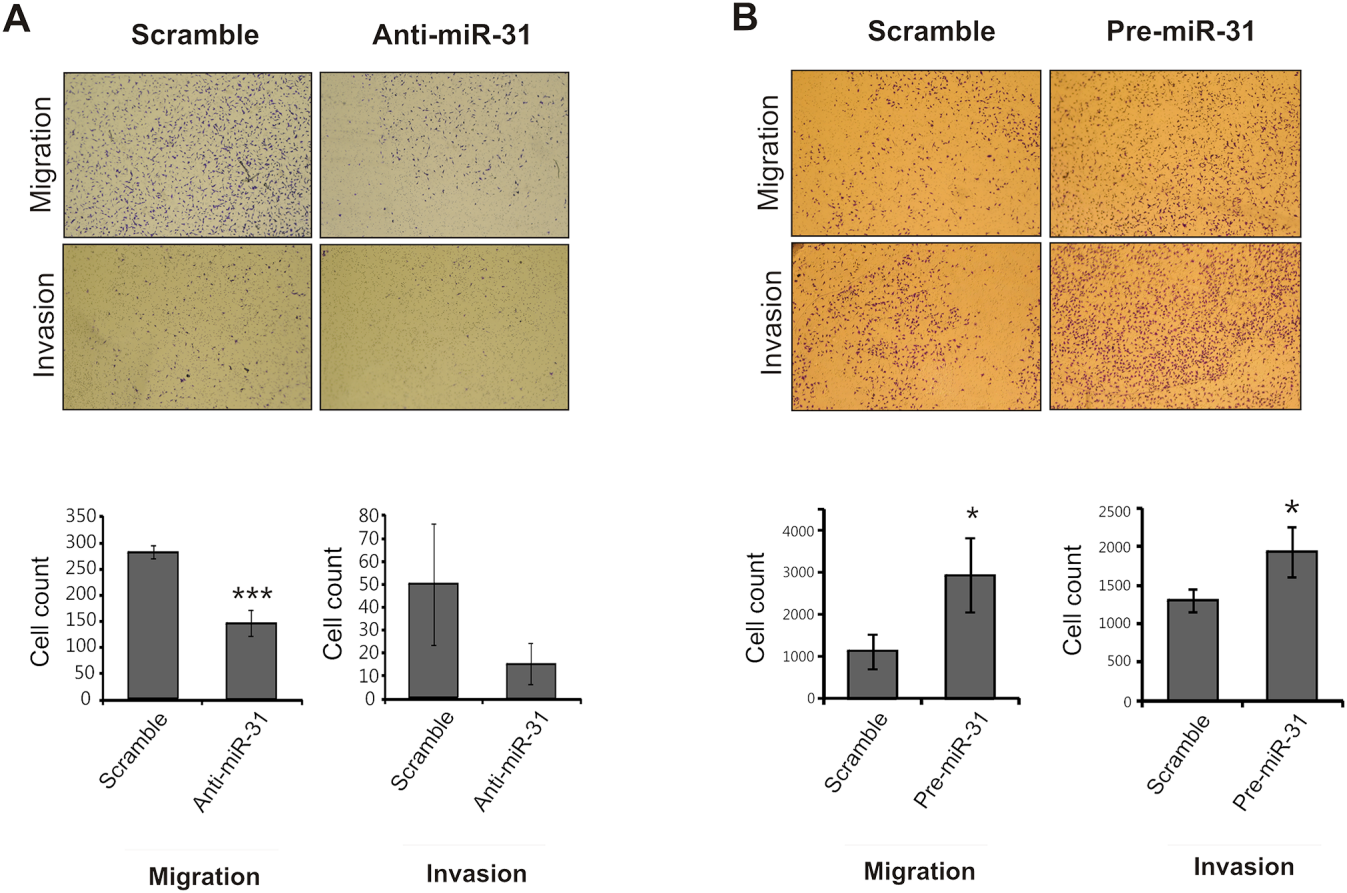
MiR-31 enhances cell migration and invasion capacity of cSCC cells. Representative photomicrographs of transwell results for UT-SCC-7 cells transfected with (A) miR-31 inhibitor (anti-miR-31) or (B) synthetic miR-31 (pre-miR-31) compared to control oligonucleotides (scramble) were taken under x100 original magnification. The number of UT-SCC-7 cells passing through the PET membrane (migration) and Matrigel (invasion) were counted. Data of one representative experiment out of three independent experiments are shown. Number of migrating and invading cells was shown in bar chart as mean ± SD value. *p<0.05; ***p<0.001, Student’s t-test.

Cell migration and invasion are crucial steps in carcinogenesis and metastasis and have been used to assess the aggressive and malignant phenotypes of the cells. Thus, we aimed to determine whether miR-31 can regulate these processes using Transwell migration and invasion assays (Figure 5). Inhibition of miR-31 in UT-SCC-7 cells strikingly reduced both migration (2-fold decrease, *p*<0.001) and invasion (5-fold decrease) of UT-SCC-7 cells (Figure 5A) demonstrating that endogenous miR-31 in UT-SCC-7 cells contributes to these processes. *Vice versa*, overexpression of miR-31 resulted in a significant increase of cell migration (3-fold increase, *p*<0.05) and invasion (1.6-fold increase, *p*<0.05) (Figure 5B). These results demonstrate the role of miR-31 in promoting the migration and invasion of cSCC cells.

### MiR-31 increases anchorage-independent colony forming of UT-SCC-7 cells

**Figure 6:**
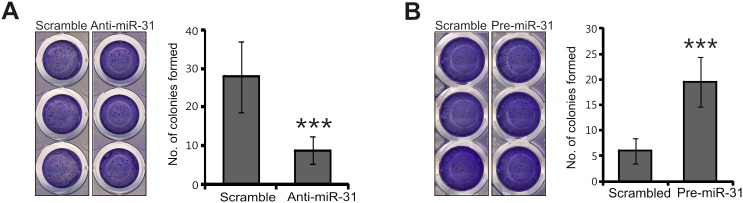
MiR-31 enhances the colony formation ability of cSCC cells. UT-SCC-7 cells were transfected with miR-31 inhibitors (anti-miR-31) or control oligonucleotides (scramble) (A); miR-31 precursor RNAs (pre-miR-31) or miRNA precursor control (scramble) (B). The colonies formed by the transfected cells were stained with crystal violet and counted 10 days after transfection. A representative experiment performed in quintuplicate is shown and this experiment was repeated three times. Number of colony was counted and presented in bar chart format as mean ± SD. ***p<0.001, Student’s t-test.

The ability of a single cell to grow into a colony in colony formation assay is a critical characteristic of tumor cells. To test whether endogenous miR-31 has a role in this process in cSCC, we performed colony formation assays with UT-SCC-7 cells. Inhibition of endogenous miR-31 decreased colony forming ability of UT-SCC-7 cells (3-fold decrease, *p*<0.001) highlighting its role in cell survival (Figure 6A). In contrast, significant increase of colony formation was observed in UT-SCC-7 cells upon ectopic overexpression of miR-31 (4-fold increase, *p*<0.001) (Figure 6B). The enhancement of colony forming ability in UT-SCC-7 by miR-31 indicated the potential roles of miR-31 in cSCC tumorigenesis.

## Discussion

In this study, we determined that miR-31 is overexpressed in cSCC and regulates several hallmarks of cancer including cell migration, invasion and colony formation. MiR-31 is among the most frequently altered miRNAs in human cancers and it has been proposed as a novel molecular target for cancer therapy and chemoprevention [11]. However, its role in tumor development is still not completely understood. Since its expression level is increased in head and neck cancer, hepatocellular carcinoma and colorectal cancer, but decreased in breast cancer, gastric cancer and prostate carcinoma, it was suggested that miR-31 can behave as either a tumor suppressor or an oncogenic miRNA, depending on the transcriptional context in which it is expressed and influenced several cancer associated phenotypes including cell growth, cell migration, metastasis and the sensitivity to chemotherapeutic agents as well [6], [16], [17]. Therefore, it is necessary to investigate the roles of miR-31 in an extended range of different tumors for a clearer view of its dynamic behavior. In this study we demonstrate the overexpression of miR-31 in cSCC, specifically in tumor cells, excluding the possibility of cellular infiltration as an explanation for miR-31 overexpression in whole-tumor samples. Interestingly, miR-31 expression was not significantly altered in actinic keratosis, the precancerous lesions which already have substantial genomic instability but which have not became invasive [2]. This implies that the increase in miR-31 expression is a late event in neoplastic evolution after formation of an invasive lesion. Supporting our findings, the overexpression of miR-31 in cSCC was previously observed reported by Sand, *et al* who performed miRNA expression profiling in cSCC tissues [18].

Our observation that endogenous miR-31 expression promotes the colony forming ability of cSCC cells indicates the importance of miR-31 in the regulation of cSCC survival. This is in agreement with previous studies showing an oncogenetic function of miR-31 in lung and oesophageal SCCs, as well as association of miR-31 with poorer relapse prognosis and patient survival in lung and oesophageal SCCs [19], [20]. The activation of anti-apoptotic and pro-survival cascades by miR-31 has also been reported in colorectal cancer via the repression of *RASA1*, a suppressor of *RAS* oncogene [16].

Our results revealed the role of miR-31 in promoting cSCC cells migration and invasion, crucial steps in tumor progression. Several important genes in which regulate cell movement and cytoskeletal have been reported to be direct target of miR-31 including *ITGA5*, *RDX* and *WAVE3* [21], [22]. Thus, it is not surprising that miR-31 possessed the fundamental role in regulating the invasion-metastasis cascade. Similar to our findings, the role of miR-31 in stimulating tumor cell migration and metastasis formation was previously reported in colorectal cancer upon inhibition of miR-31 [16]. In contrast, down-regulation of miR-31 in primary breast tumor was associated with metastasis [23], indicating a complex role for miR-31 in cancer, that could be dependent on cancer type and tumor stage. Although miR-31 is typically down-regulated in primary breast tumors, high miR-31 levels were detected in plasma samples of breast cancer patients, and also in patients with other cancer types such as oral carcinoma. These suggested the potential application of miR-31 as a diagnostic marker [24]. The diagnostic potential of circulating miR-31 in cSCC remains to be determined.

In conclusion, our results demonstrate that high endogenous level miR-31 in cSCC cells promote their migration, invasion and colony forming ability, and that inhibition of miR-31 can suppress these phenotypes. These results indicate the importance of miR-31 in cSCC hallmarks and its potential as a target for cSCC treatment.
